# Systematic Review of the Relationship between Acute and Late Gastrointestinal Toxicity after Radiotherapy for Prostate Cancer

**DOI:** 10.1155/2015/624736

**Published:** 2015-11-30

**Authors:** Matthew Sean Peach, Timothy N. Showalter, Nitin Ohri

**Affiliations:** ^1^Department of Radiation Oncology, University of Virginia School of Medicine, Charlottesville, VA 22908, USA; ^2^Department of Radiation Oncology, Montefiore Medical Center, Albert Einstein College of Medicine, Bronx, NY 10467, USA

## Abstract

A small but meaningful percentage of men who are treated with external beam radiation therapy for prostate cancer will develop late gastrointestinal toxicity. While numerous strategies to prevent gastrointestinal injury have been studied, clinical trials concentrating on late toxicity have been difficult to carry out. Identification of subjects at high risk for late gastrointestinal injury could allow toxicity prevention trials to be performed using reasonable sample sizes. Acute radiation therapy toxicity has been shown to predict late toxicity in several organ systems. Late toxicities may occur as a consequential effect of acute injury. In this systematic review of published reports, we found that late gastrointestinal toxicity following prostate radiotherapy seems to be statistically and potentially causally related to acute gastrointestinal morbidity as a consequential effect. We submit that acute gastrointestinal toxicity may be used to identify at-risk patients who may benefit from additional attention for medical interventions and close follow-up to prevent late toxicity. Acute gastrointestinal toxicity could also be explored as a surrogate endpoint for late effects in prospective trials.

## 1. Introduction

Prostate cancer is the most commonly diagnosed noncutaneous malignancy in men in developed countries. Definitive external beam radiotherapy (RT) is a treatment option for the majority of patients who present with localized disease. Additionally, RT may be offered after radical prostatectomy for patients whose pathology demonstrates adverse pathologic features or as salvage therapy for recurrent disease after surgery.

Regardless of the treatment technique used, RT for prostate cancer exposes a portion of the lower gastrointestinal (GI) tract to ionizing radiation and consequently carries a risk of GI toxicity. GI toxicity is categorized as occurring within two possible phases: acute (typically within 3 months of treatment) and late (more than 3 months after treatment) [1, 2]. Symptoms can range from a mild increase in bowel movement frequency to more severe complications such as rectal bleeding, pain, or fistula. The acute phase of RT injury is characterized by inflammation in response to therapy, while the late phase is characterized by fibrosis and sclerosis within the GI tract [3]. While mild to moderate acute GI toxicity is more common than late toxicity, the potential permanent impact of late GI toxicity is thought to bear more clinical significance.

In an analysis of 35 studies including nearly 12,000 patients, rates of moderate (generally grade ≥ 2) and severe (grade ≥ 3) late GI toxicity following definitive external beam RT for prostate cancer were 15% and 2%, respectively [4]. Dose-escalated RT, which has been shown to improve disease control rates [5] and is now standard of care, increases the risk of late GI toxicity [4–7]. Reported rates of late GI toxicity appear to be decreased when dose-escalated RT is delivered using advanced treatment techniques such as intensity-modulated radiotherapy (IMRT) [4]. Other measures to limit GI toxicity, such as the administration of radioprotective medications [8] or the use of spacers to separate the prostate and rectum [9], are being explored. Randomized clinical trials, with large numbers of patients and lengthy follow-up, will be required to establish the efficacy of these toxicity prevention strategies.

There may be a consequential relationship between temporary acute GI toxicity and permanent late GI toxicity in prostate cancer patients treated with RT [10]. In this paper, we perform a systematic review to characterize the relationship between acute and late GI complications from prostate RT. We detail mechanisms by which acute toxicity may lead to consequential late effects. Finally, we explore the possibility of exploiting this connection for the identification of patients at risk of late GI toxicity and for the development of novel clinical trials for toxicity prevention.

## 2. Methods

### 2.1. Study Selection

We searched PubMed (http://www.ncbi.nlm.nih.gov/pubmed/) on May 1, 2013, for the terms “radiation therapy”, “late”, “early”, and “side effects”, with no limits placed on publication date. Duplicates and non-English language articles were excluded, and abstracts of all remaining manuscripts were read. Articles focusing on acute and late toxicity from external beam RT for prostate cancer were selected and examined in detail. Studies that examined the potential relationship between acute and late GI sequelae prostate RT were included in the final analysis. When two reports seemed to describe overlapping patient populations, the most recent publication was utilized for this analysis.

### 2.2. Data Extraction and Clinical Endpoints

Data abstraction was conducted according to the Preferred Reporting Items for Systematic Reviews and Meta-analyses (PRISMA) statement [11]. For each study, the following information was extracted: name of the first author, year of publication, name of the clinical trial (if applicable), sample size, and RT protocol. The primary measure of interest for this analysis was the association between late GI toxicity and acute GI toxicity. Hazard ratios, risk ratios, correlation coefficients, and other statistical measures describing the relationship between acute and late GI toxicity were extracted. Our preliminary analysis indicated that available data were not appropriate for meta-analysis, so we proceeded to perform a PRISMA-style systematic review.

## 3. Results

### 3.1. Study Selection

**Figure 1:**
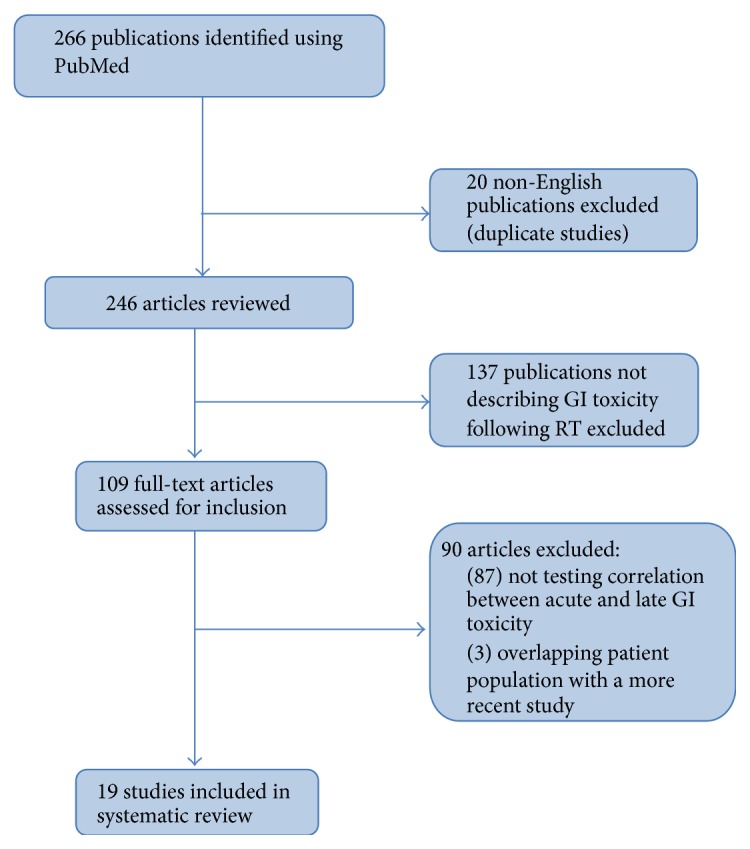
Selection strategy for systematic review of the published literature evaluating the relationship between acute and late gastrointestinal toxicity following prostate radiation therapy.

Search results are summarized in Figure 1. Our initial search yielded 266 results. Removal of duplicates and non-English language manuscripts reduced this number to 246. 109 papers met initial eligibility criteria and were read at full length to determine if statistical tests for a link between acute and late GI toxicity were reported. Most of the papers were eliminated for merely reporting rates of acute and/or late GI toxicity in the study population. Others were excluded because they combined GI and genitourinary effects in their analyses. Three papers described patient populations that were likely included in subsequent reports from the same institutions. In total, 19 manuscripts met all eligibility requirements and were included in this report.

Ten manuscripts reported results from prospective trials [12–21] (Table 1), and nine were retrospective reviews of institutional experiences or databases [22–30] (Table 2). The trials that examined acute and late side effects involved a variety of techniques including conventional prostate RT [19], 3D conformal RT (3DCRT) [14, 15, 17, 24, 25, 30], high dose intensity modulated RT (IMRT) [27], hypofractionated IMRT [20, 23], combination brachytherapy + IMRT [29], and a mixed population treated with IMRT and 3DCRT [28]. Comparisons were also made in the manuscripts between different techniques including 3DCRT versus conventional RT [18]; 3DCRT dose escalation versus standard dose RT [12, 21]; 3DCRT versus hypofractionated 3DCRT [13]; a varied experience of 3DCRT and conventional RT ± ADT [22]; high dose IMRT versus high dose 3DCRT [26]; and high dose IMRT ± whole pelvis RT [16] (Table 1). The primary objective of most papers was to examine clinical factors leading to the development of acute GI and late GI toxicity [12–14, 16–18, 22, 28, 30], while some specifically focused on the relationship between acute and late side effects [14, 19, 21, 30]. The goal of other manuscripts was to simply report acute and late effects of a particular treatment [15, 20, 21, 23–27, 29]. Some of these studies tested medications to decrease toxicity, including rectal prostaglandin administration [15] and rectal sucralfate [19].

### 3.2. Evidence of Association between Acute and Late GI Toxicity

The manuscripts specifically looking for associations between acute and late effects demonstrated mild to strong correlations in various aspects of GI toxicity. Pinkawa et al. reported that acute bowel bothersome scores were associated with poor long-term bowel bothersome scores on univariate analysis, although with a HR of only 1.05; this relationship was not statistically significant in a multivariate analysis that accounted for RT dose and volume parameters [14]. Moderate to strong associations were found between multiple aspects of acute and late GI toxicity by Heemsbergen et al., where multivariable analysis suggested minimal contribution of RT dose and volume effects [21]. Acute proctitis was strongly (HR 2.9) associated with long-term diarrhea, defined as ≥6 stools a day. Acute mucosal discharge was predictive of later use of incontinence pads (HR 2.1). Interestingly, the authors note that more objective factors of GI toxicity such as bleeding not included in RTOG had a stronger correlation. This suggests that a different scoring scheme may better demonstrate consequential GI toxicity relationships [21]. Perhaps the strongest evidence of this relationship came from O'Brien et al., who found that grade ≥2 acute rectal pain was associated with grade ≥2 late rectal toxicity with a HR of 3.4 [19]. However this manuscript did not examine dosimetric parameters, so observations were not adjusted for potential RT dose-volume interactions.

The strongest findings attesting to the relationship of acute and late GI toxicity came from manuscripts not primarily interested in determining this effect. Zelefsky et al., while specifically looking at late toxicity response to 3DCRT dose escalation in 1571 participants, found that acute grade ≥2 GI toxicity was a strong predictor of late grade ≥2 GI toxicity (HR 6.95, *p* < 0.001). There was little contribution from dosimetric factors on multivariate analysis [28]. A similarly strong relationship was found between acute grade ≥2 proctitis and late grade ≥1 GI toxicity (OR 6.05, *p* = 0.03) in a trial looking at the effect of misoprostol suppository on late rectal toxicity following 3DCRT [15]. Further, multivariate examination of late GI toxicity in over 100 patients subjected to high dose 3DCRT demonstrated that grade ≥2 acute fecal incontinence was associated with chronic/late grade ≥2 fecal incontinence (OR 4.43, *p* = 0.004) and a stronger relationship for acute grade 3 fecal incontinence (OR 6.9, *p* = 0.001) [17]. Of the remaining papers, most of the correlations were mild to moderate or were not significant once placed in multivariate analysis with dosimetric considerations (Tables 1 and 2).

### 3.3. Mechanism for Associations

With some exceptions [13, 18, 19, 21, 30], most of the manuscripts that demonstrated an association between acute and late toxicities did not delve into the mechanism behind their findings. Koper et al. briefly discussed that their findings were most likely the result of consequential effects [18], namely, that acute toxicity leads to inflammation, leading to leakage of intestinal contents and eventual fibrosis manifesting as late toxicity. Alternatively, Arcangeli et al. concluded that the association between acute and late toxicity was evidence for a consequential mechanism but instead was a result of shared dosimetric risk factors. Interestingly, the association between acute and late effects observed in their conventionally fractionated RT arm was not observed in their hypofractionated RT arm [13].

Complementing their thorough data analysis, Heemsbergen et al. provided a more comprehensive explanation of the observed relationship between acute and late toxicity [21]. The authors put forth two possible mechanisms: the first a simple dose-volume relationship, and the second a continuum of consequential damage as has been observed in animal models [31]. In the former mechanism, severity of acute and late GI toxicity may correlate due to independent dose-volume effects on the RT at each time frame. Having the benefit of a large patient population, adequate follow-up, and uniform characterization of acute and late toxicity, the authors found on multivariate analysis that acute toxicity remained independently associated with late effects after adjusting for dosimetric variables and concluded that the relationship was most likely a combination of consequential effects and some dose volume effects. Of note, the group indicated that the acute RTOG score was not the most correlative factor; rather, tracking of individual GI symptoms was more revealing of the consequential pattern [21].

Heemsbergen indicated that other studies looking at acute-late correlation suffered from a lack of dose-volume considerations in their analysis [21]. This included the work of O'Brien et al. whose authors concede to not including dosimetric data but attest to dose-volume effects not contributing to acute toxicity in another study of a similar population and technique [19]. Koper et al. [18] offer alternative explanations to the observed correlation including inherent properties in individuals, such as yet described genetics or comorbidities that lead to greater tissue sensitivity to radiation both acutely and chronically. A different explanation offered by Heemsbergen et al. is that some patients are more likely to communicate their symptoms and thus will verbalize both acute and late side effects alike [21]. However, the authors felt this theory was unlikely, as more objective findings such as acute and late rectal bleeding demonstrated strong associations [21]. The article by Jereczek-Fossa et al. was the most recent manuscript to thoroughly expand upon a proposed mechanism behind their demonstrated acute-late GI toxicity association [30]. The group believed, given the lack of prostate dose influence on late toxicity in a cohort of nearly 100 patients, that the association stemmed from consequential effects initiated by damage to the GI mucosa in the acute phase, citing works looking at consequential rectal toxicity in cervical cancer. Additionally, the authors asserted that age factored into the development of consequential damage when applied to multivariable analysis, in particular affecting acute toxicity as a result of comorbidities or an indirect effect of treatment decision-making [30].

Although not identified in the literature review, we are aware of the work by Campostrini et al. that showed more robust evidence of pathological consequential toxicity in humans [32]. Their group followed the progress of 130 patients from immediately after prostate RT and throughout the late period endoscopically (median follow-up of 84 months). It was noted that acute damage affected both the rectum and the anal canal macroscopically, with the most notable finding of hemorrhoid congestion, which was a major contributor to acute rectal bleeding. Interestingly, two patients had acute proctoscopic findings that were not manifested clinically. The finding of clinical and/or proctoscopic acute proctitis was strongly predictive of late toxicity (HR 5.6. 95% CI 2.1–15.2, *p* = 0.001) on multivariate analysis that incorporated dosimetric parameters [32].

### 3.4. Difficulties in Reporting the Correlation between Acute and Late Toxicities

Some of the authors of the manuscripts that did not observe a correlation between acute and late toxicities commented on the lack of findings, when viewed from the perspective of contradictory findings. Ballar et al. attributed the observed lack of correlation to a lower than normal acute toxicity found in their particular study compared to others [25]. Similarly, Zelefesky and colleagues manuscript did not identify a relationship between acute and late toxicities after combination brachytherapy and external beam RT, and they stated that this was likely due to fewer acute and late side effects than found in most similar studies [29]. Likewise, some manuscripts that did observe a relationship between acute and late toxicities also indicated potential study shortcomings that would underestimate the true consequential effect, such as short follow-up and newer RT techniques including IMRT that may cause less severe toxicity in both the acute and late setting [30]. Jereczek-Fossa et al. also noted that retrospective studies may suffer from underreported acute and late GI toxicity rates, complicated by the complexity of reporting late term side effects in prostate RT [30]. It has also been suggested that physician-based toxicity scoring, rather than patient-directed assessments, may introduce significant reporting bias [23].

## 4. Discussion

### 4.1. Acute Toxicity Is Predictive of Late Toxicity

The heterogeneity of the available data precludes quantitative synthesis in a formal meta-analysis, but we believe that the findings from this review shed significant light on the relationship between acute and late GI complications from prostate RT. Thirteen of the 19 studies that met inclusion criteria demonstrated an association between acute and late complications. Reports where no such associations were found tended to have a significantly smaller sample size than “positive” studies. Restricting our analysis to series with at least 200 subjects, for example, would leave nine remaining studies, all of which report a statistically significant link between acute and late complications. We therefore conclude that the overwhelming majority of the published evidence supports the presence of an association between acute and late GI toxicity following RT for prostate cancer.

Campostrini et al. provided strong evidence of pathologically confirmed acute toxicity as a significant predictor of late GI toxicity, even when dosimetric parameters as well as RT technique are taken into account. These findings are further bolstered by animal studies showing a stepwise pathologic progression from acute to late effects [33, 34]. Therefore, the trends found in this systematic review of clinical studies, combined with observations from animal models, support a second important conclusion: acute toxicity may serve as an appropriate surrogate for late GI toxicity as a way to identify patients at high risk of developing permanent late GI toxicity, potentially for clinical trials of novel therapies intended to prevent development of consequential late GI toxicity, and as a surrogate endpoint for clinical trials.

### 4.2. Significance of Consequential Effect for Research

Since clinically significant late GI toxicity occurs in a minority of patients, clinical trials of medical interventions designed to prevent GI toxicity after prostate RT may require an excessively large sample size, if the trial designs are such that any prostate RT patient is eligible. However, establishing a consequential relationship between acute and late GI toxicity presents the opportunity to develop more efficient trial designs by focusing on a high-risk population. If acute GI toxicity is used as an eligibility criterion, which would restrict the study population to those at highest risk of late GI toxicity, a candidate medical intervention could be studied in clinical trial with a higher likelihood of identifying an effective strategy to reduce late GI toxicity. For example, if the study population has a 40% risk of late GI toxicity after IMRT (based on including only patients with significant acute toxicity), then a randomized, controlled trial of intervention versus placebo with a sample size of just 100 subjects would have 71% power to detect a 50% reduction in late toxicity events (personal communication, Nolan Wages, Ph.D.). If the baseline toxicity risk was 10% and all other parameters were unchanged, the study would only have a power of 25%. Notably, this hypothetical example is potentially realistic, based on the available evidence identified in this report: assuming a 15% average risk of grade 2 or higher late GI toxicity [4] and a three- to sixfold increase in rates of late GI toxicity among patients with grade 2 or higher acute GI toxicity [19, 21, 28], the risk of late toxicity among those patients with acute toxicity would be at least 40% and likely much higher.

The consequential nature of GI toxicity could be therefore exploited in research trials looking at interventions to avoid late toxicity in a number of ways:

1. In future studies, acute toxicity may be used as a surrogate endpoint for late toxicity. This could decrease the duration and sample size required for prospective trials.
2. Patients who demonstrate acute toxicity can be selected for long-term studies of late toxicity.
3. Previously treated cohorts for which acute toxicity data are recorded can be assessed selectively for late toxicity, sampling only those patients who demonstrated acute GI toxicity.

### 4.3. Future Potential Studies: Pharmacological

With the above study framework, there are a number of pharmacological and nonpharmacological interventions than can be explored, some of which have already demonstrated the ability to prevent GI toxicity in pelvic radiation. This topic has been detailed in several recent reviews [35–37]. In regard to pharmacological interventions, the Cochrane review by Ali and Habib best summarizes the available options including such interventions as aminosalicylic acid (ASA) derivatives, sucralfate, arginine, vitamin E, probiotics, misoprostol, short chain fatty acid enemas, corticosteroids, cholestyramine, vitamin A, estrogen/progesterone, and octreotide [38]. Highlights from that review included manuscripts demonstrating the prevention of acute GI toxicity with oral sulphasalazine in prostate RT [39], as well as a decrease in acute and late GI toxicity when oral sucralfate is applied during and after RT for prostate and bladder cancer [40]. However, the previously cited work [19] and an earlier manuscript [41] by O'Brien et al. counter these findings when sucralfate is applied as a suppository during prostate RT in studies of similar size. Lastly, Ali and Habib referenced a small study showing that misoprostol suppositories applied before prostate external beam RT had protective properties [38]. Again, works encountered in this systematic review with larger numbers of participants did not find any benefit of misoprostol suppositories in the acute and late term [15].

There are other promising potential pharmacological compounds for preventing GI toxicity during pelvic RT. In regard to ASA derivatives, patients randomized to oral balsalazide during prostate RT achieved a CTC v2.0 prostate index of 35.3 versus 74.1 in placebo at two weeks after therapy (*p* = 0.04) [42]. However, a trial arm examining the rectal application of the ASA derivative mesalazine was prematurely terminated because of increased acute toxicity during prostrate 3DCRT in comparison to sucralfate enema control (HR 2.5, 95% CI 1.1–5.7, and *p* = 0.03) [43]. In the same study, no difference was found between sucralfate enema and hydrocortisone enema in preventing acute rectal toxicity [43]. Intrarectal application of the steroid beclomethasone in a more recent placebo controlled study did demonstrate an improved irritable bowel disease quality of life index, less rectal bleeding, and superior Vienna Rectoscopy Score up to 12 months following prostate RT [44]. Hyaluronic acid suppositories have also demonstrated the ability to decrease and delay acute radiation proctitis, according to RTOG scoring, when compared to historical prostate RT controls [45].

The free radical scavenger amifostine, when administered regularly during RT, has shown great potential in preventing acute rectal toxicity. In a randomized trial of 36 patients undergoing a mix of pelvic RT, intrarectal amifostine resulted in a significant decrease in RTOG score (*p* < 0.001), a decrease in LENT-SOMA score (*p* = 0.002), and improved proctoscopic tissue examination compared to controls [46]. More recent work has shown that increasing the dose of amifostine results in greater reduction of GI toxicity as determined by the EPIC bowel bothersome score during treatment and at 12 months after external prostate RT [47]. Lastly, the feasibility of rectal injections of Botox as a preventative measure against acute rectal toxicity has recently been studied based on Botox's effect on muscle spasticity, with future efficacy trials planned [48]. In total, there are a great deal more pharmaceutical compounds that have shown success in treating acute GI toxicity than treating late GI toxicity or preventing acute or late GI toxicity. Reassessment of some of these compounds as outlined above may be able to tease out greater usefulness in the above and other compounds.

### 4.4. Future Studies: Nonpharmacological

In regard to nonpharmacological strategies to prevent GI toxicity from pelvic RT, rectal balloons have been studied for a number of years and are used routinely in some practices, particularly in proton beam RT. However, there appears to be only one small work to show decreased late GI toxicity, compared to treatment without a rectal balloon, as determined by proctoscopic assessment at two-year follow-up [49]. Rectal balloons have shown good patient compliance and tolerance [50]. The use of injectable spacers is an alternative approach that creates space between the prostate target volume and the rectum. For example, prospective evaluation with a polyethylene glycol hydrogel spacer in small study of 10 patients demonstrated very low acute GI toxicity. [51]. Collagen injections have also been used to increase prostate-rectal distance, resulting in a 50% decrease in the RT dose to the rectum [52]. Recently, a hybrid idea that in simulation appears to function well is the biodegradable interstitial balloon [53]. In comparison to some of the pharmacologically based preventative measures, studies of spacer interventions are for the most part lacking assessment on late term effects and would greatly benefit from study designs where acute toxicity was applied as a surrogate for late toxicity or used for patient selection in long-term trials.

## 5. Conclusions

Published data strongly support the presence of an association between acute and late GI toxicity following RT for prostate cancer. We suggest that acute GI toxicity may be used by physicians to identify patients who may benefit from personalized counseling and supportive care to address a high risk of permanent late GI toxicity. Furthermore, trials of strategies to prevent late morbidity might be enhanced by the preferential enrollment of subjects who develop acute toxicity in order to evaluate potential preventive strategies in a cohort of patients at high risk of late toxicity.

## Figures and Tables

**Table 1:** Summary of prospective manuscripts studying relationship of acute and late GI toxicity after prostate RT.

Study name [citation]	Study design	Toxicity analysis time points		Toxicity grading system		Follow-up duration	Acute & late toxicity correlation
		Acute end	Late start	Acute	Chronic		
Medical Research Council RT01 trial, Barnett et al. 2011 [12]	(i) Arm 1: 74 Gy/37 F (*n* = 394) 3DCRT (ii) Arm 2: 64 Gy/32 F (*n* = 394) 3DCRT	6 W PTC	2 Y PTC	Acute RTOG	Late RTOG, LENT/SOMA, UCLA-PCI, RMH	Median not reported (2–5 Y)	Yes

Arcangeli et al. 2011 [13]	(i) Arm 1: 80 Gy in 40 F (*n* = 85) 3DCRT (ii) Arm 2: 62 Gy in 20 F (*n* = 83) 3DCRT	1 M PTC	6 M PTC	Modified acute RTOG	Modified LENT/SOMA	(i) Arm 1 median 32 M (8–66 M) (ii) Arm 2 median 35 M (7–64 M)	Yes

Pinkawa et al. 2010 [14]	70.2 or 72 Gy in 1.8–2.0 Gy/F (*n* = 298), 3DCRT	6 M PTC	12 M PTC	Expanded Prostate Cancer Index Composite (EPIC)	Expanded Prostate Cancer Index Composite (EPIC)	Median 16 M (12–20 M)	Yes

Kertesz et al. 2009 [15]	(i) 60–72 Gy, 1.8–2 Gy/F, (*n* = 100), 3DCRT (ii) Unreported number on ADT	TRT	Assume 90 D PTC	CTC v2	RTOG, LENT/SOMA	Median 50 M (9–59 M)	Yes

Guckenberger et al. 2010 [16]	(i) 76.23 Gy/33 F (*n* = 74) IMRT (ii) 73.91 Gy/32 F (*n* = 26) post prostatectomy IMRT	6 W PTC	6 M PTC	CTCAE v3.0	CTCAE v3.0	Median 26 M	Yes

AIROPROS 0102, Fellin et al. 2009 [17]	70 Gy, 1.8–2 Gy/F (*n* = 718), 3DCRT	1 M PTC	6 M PTC	Custom fecal incontinence and bleeding questionnaire	Custom fecal incontinence and bleeding questionnaire	Median 36 M	Yes

Koper et al. 2004 [18]	(i) 66 Gy in 2 Gy/F (*n* = 123) 3DCRT (ii) 66 Gy in 2 Gy/F (*n* = 125) Conventional (iii) 15% got adjuvant ADT	Assume 90 D PTC	1 Y PTC	Acute RTOG, modified Tait, and Fransson questionnaire	Late RTOG, modified Tait, and Fransson questionnaire	Median not reported, 93% followed to 2 Y	Yes

Heemsbergen et al. 2006 [21]	(i) 68 Gy in 2 Gy/F (*n* = 275) 3DCRT (ii) 78 Gy in 2 Gy/F (*n* = 278) 3DCRT	28 to 120 D PTC	120 D PTC	Acute RTOG, maximum score of acute mucous discharge	Late RTOG	Median 44 M	Yes

Trans-tasman radiation oncology group, O'Brien et al. 2002 [19]	52.5 Gy in 20 F (*n* = 23) or 63–65 Gy in 2 Gy/F fractions (*n* = 63), conventional	Assume 90 D PTC	Assume 90 D PTC	Assume RTOG/EORTC	RTOG/EORTC	Median 63 M	Yes

Goineau et al. 2013 [20]	(i) 76 Gy in 38 F (*n* = 38), IMRT (ii) *n* = 21 ADT (6 M to 3 Y)	2 M PTC	6 M PTC	CTCAE V3, QLQ-C30, and QLQ-PR25	CTCAE V3, QLQ-C30, and QLQ-PR25	54 M	No

PTC = posttreatment completion, univariate (UV), multivariate (MV), androgen deprivation therapy (ADT), and TRT = throughout radiotherapy.

Acute RTOG = Radiation Therapy Oncology Group (RTOG) and the European Organization for Research and Treatment of Cancer acute morbidity rating scale.

Late RTOG = Radiation Therapy Oncology Group (RTOG) and the European Organization for Research and Treatment of Cancer late morbidity rating scale.

LENT = Late Effects Normal Tissue Task Force scale.

SOMA = Subjective, Objective, Management, and Analytic (SOMA) scales.

UCLA-PCI = University of California Loss Angeles Prostate Cancer Index.

RMH scale = Royal Marsden Hospital scale.

EPIC = Expanded Prostate Cancer Index Composite.

CTC v2 = Common Toxicity Criteria v2.0.

CTCAE V3 = Common Terminology Criteria for Adverse Events v3.0.

QLQ-C30 = EORTC QLQ-C30 quality of life questionnaire.

QLQ-PR25 = EORTC QLQ-PR25 quality of life questionnaire.

WHO = World Health Organization criteria.

**Table 2:** Summary of retrospective manuscripts studying relationship of acute and late GI toxicity after prostate RT.

Study name [citation]	Study design	Toxicity analysis time points		Toxicity grading system		Follow-up duration	Acute/late GI toxicity association
		Acute end	Late start	Acute	Chronic		
Zilli et al. 2011 [23]	(i) IMRT 56 Gy in 4 Gy/F (*n* = 82) (ii) Neoadjuvant ± concurrent ADT (*n* = 12)	6 W PTC	6 M PTC	Acute RTOG	Late RTOG	Median 48 M (9–67 M)	No

Fiorica et al. 2010 [24]	(i) 78 Gy in 2 Gy/F, 3DCRT (*n* = 26) (ii) 78 Gy in 2 Gy/F + 6 M AST, 3DCRT (*n* = 81)	TRT	3 M PTC	WHO	SOMA	Median 35 M (9–88 M)	No

Ballar et al. 2009 [25]	74 Gy in 2 Gy/F, 3DCRT (*n* = 104)	6 M PTC	6 M PTC	Acute RTOG	Late RTOG	Median 30 M (20–50 M)	No

Shu et al. 2001 [26]	72.0 to 79.2 Gy, 3DCRT (*n* = 26) or IMRT (*n* = 18)	6 M PTC	6 M PTC	Acute RTOG	Late RTOG	Median 23.1 M (10–84.7 M)	No

Cahlon et al. 2008 [27]	(i) 86.4 Gy/48 F IMRT (*n* = 478) (ii) Some had adjuvant 3–6 M ADT	90 D PTC	90 D PTC	CTCAE V3	CTCAE V3	Media 53 M	Yes

Zelefsky et al. 2008 [28]	(i) 66–81 Gy, 1.8 Gy/F, 3DCRT or IMRT (*n* = 1571) (ii) Neoadjuvant ADT 3 M (*n* = 678)	Assume 90 D PTC	Assume 90 D PTC	Assume CTCAE V3	CTCAE V3	Median 8 Y (5–18 Y)	Yes

Jereczek-Fossa et al. 2010 [30]	(i) Definitive RT 76 Gy in 2 Gy/F, 3DCRT (*n* = 542) (ii) Postprostatectomy RT 70 Gy in 2 Gy/F, 3DCRT (*n* = 431)	3 M PTC	3 M PTC	Acute RTOG	Late RTOG	Median 25.2 M (1–129 M)	Yes

Liu et al. 2004 [22]	(i) Prospective database (*n* = 1192) (ii) 52.5–72 Gy in 20–36 F, conventional or 3DCRT (iii) Neoadjuvant (*n* = 459), median duration (5.1 M), concurrent (*n* = 285), adjuvant (*n* = 222), and median duration (11 M)	Assume 90 D PTC	Assume 90 D PTC	Assume Acute RTOG	Modified RTOG/SOMA	Median 49 M (24–105 M)	Yes

Zelefsky et al. 2008 [29]	I^25^ implantation (110 Gy) followed in 2 M by 50.4 Gy of IMRT in 1.8 Gy/F (*n* = 127)	90 D PTC	90 D PTC	CTCAE	CTCAE	Median 30 M	No

PTC = posttreatment completion, univariate (UV), multivariate (MV), androgen deprivation therapy (ADT), and TRT = throughout radiotherapy.

Acute RTOG = Radiation Therapy Oncology Group (RTOG) and the European Organization for Research and Treatment of Cancer acute morbidity rating scale.

Late RTOG = Radiation Therapy Oncology Group (RTOG) and the European Organization for Research and Treatment of Cancer late morbidity rating scale.

LENT = Late Effects Normal Tissue Task Force scale.

SOMA = Subjective, Objective, Management, and Analytic (SOMA) scales.

UCLA-PCI = University of California Loss Angeles Prostate Cancer Index.

RMH scale = Royal Marsden Hospital scale.

EPIC = Expanded Prostate Cancer Index Composite.

CTC v2 = Common Toxicity Criteria v2.0.

CTCAE V3 = Common Terminology Criteria for Adverse Events v3.0.

QLQ-C30 = EORTC QLQ-C30 quality of life questionnaire.

QLQ-PR25 = EORTC QLQ-PR25 quality of life questionnaire.

WHO = World Health Organization criteria.
